# Preliminary study on the diagnosis of NK stress based on the puncture mechanical characteristics of cucumber stem

**DOI:** 10.1186/s12870-023-04675-0

**Published:** 2024-01-03

**Authors:** Yong Liu, Yafei Wang, Bin Wang, Qiang Shi, Hanping Mao

**Affiliations:** 1 School of Intelligent Agriculture, Suzhou Polytechnic Institute of Agriculture, Suzhou, Jiangsu Province China; 2 School of Agricultural Engineering, Jiangsu University, Zhenjiang, Jiangsu Province China; 3 School of Science and Technology, Shanghai Open University, Shanghai, China

**Keywords:** Greenhouse cucumber, Mechanical characteristics, Nutrient stress, Puncture, Stem

## Abstract

To investigate the relationship between stem puncture mechanical characteristics and NK stress diagnosis, the microstructure, surface morphology, cellulose and lignin content, puncture mechanical characteristics, and epidermal cell morphology of cucumber stems were measured herein. The results indicated that the middle stem, which had a diameter of approximately 7000 μm, was more suitable for puncturing due to its lower amount of epidermal hair, and its gradual regularity in shape. Further, the cucumber stems were protected from puncture damage due to their ability to rapidly heal within 25 h.. The epidermal penetration of the cucumber stems increased with the increase in cellulose and lignin, though cellulose played a more decisive role. The epidermal break distance increased with an increase in N application and decreased with an increase in K^+^ application, but the change in intercellular space caused by K^+^ supply was the most critical factor affecting the epidermal break distance. In addition, a decrease in K^+^ concentration led to a decrease in epidermal brittleness, whereas the factors affecting epidermal toughness were more complex. Finally, we found that although the detection of epidermal brittleness and toughness on nutrient stress was poor under certain treatment, the puncture mechanical characteristics of the stem still had a significant indicative effect on N application rate. Therefore, elucidating of the relationship between the puncture mechanical characteristics of the stems and crop nutritional stress is not only beneficial for promoting stem stress physiology research but also for designing on-site nutritional testing equipment in the future.

**Supplementary Information:**

The online version contains supplementary material available at 10.1186/s12870-023-04675-0.

## Introduction

Cucumber is a highly popular vegetables worldwide [1, 2]. However, current levels of nitrogen (N) fertilizer have led to low fertilizer utilization efficiency and environmental damage and have also increased the nitrate content of fruits, thereby potentially breaching the safety and hygiene standards of Chinese vegetables [3, 4]. There is a close relationship between potassium (K) and N, and higher K^+^ application rates significantly promote the absorption, transport, and reduction of NO^−^_3_ in leaves [5, 6]. In addition, K fertilizer promotes the utilization efficiency of N fertilizer, and K^+^ enhances the transport of organic N compounds to the fruit, thereby improving the nutritional value of cucumber fruit [7, 8]. Therefore, studying rational fertilization through the precise analysis of the response of cucumber to NK nutrient stress has received widespread attention.

Biological and mechanical principles have been used to assess the mechanical characteristics of crop stems [9]. The texture analyzer puncture test method has various advantages, including that is rapid, produces timely information, and inflicts only a small wound size. It has also been widely used in the identification and analysis of fruit quality [10, 11] or the determination of stem epidermal penetration in crop lodging resistance research [12, 13]. Studies have shown that water and fertilizer conditions alter the mechanical characteristics of crop stems, such as shear and tension, by affecting their component content and tissue structure [14–17]. However, these previous studies were mostly aimed at assisting in the design of agricultural machinery or exploring the lodging resistance characteristics of crops. Research on stem stress physiology is lacking, especially the changes in stem mechanical characteristics under different N and K conditions, as well as the use of stem mechanical characteristics to analyze crop health. Therefore, studying the puncture mechanical characteristics of the stem in response to water and fertilizer stress and establishing a diagnostic method for N and K nutrition in greenhouse cucumbers have broad application prospects.

Based on previous research, and given the lack of NK nutrition diagnosis methods for greenhouse cucumber, this article investigates the relationship between stem mechanical characteristics and NK stress. The findings promote the application of stem characteristics in crop stress physiology research, encouraging the use of stems in NK stress nutrition diagnosis. Moreover, this study also provides a theoretical basis for the establishment of NK nutrition diagnosis methods based on stem mechanical characteristics, as well as for the further development of relevant equipment for on-site nutritional assessment.

## Materials and methods

### Test location and details

The experiment was conducted in a Venlo-type greenhouse at the Key Laboratory of Modern Agricultural Equipment and Technology of the Ministry of Education, Jiangsu University from February 2023 to May 2023. The research object was selected "Jinyou No.1" cucumber cultivated by Tianjin Academy of Agricultural Sciences. Seedlings were raised in a plastic hole tray on February 12, 2023, and cucumber seedlings with true leaves were transplanted into a plastic basin filled with 8L of perlite on February 26.

### Experimental design

The experiment was performed according to a previous study [18] with a small modification. The grouping treatment was performed on the 10th day after transplantation. The experimental setup included nine treatment groups, each containing 20 cucumber plants. Data collection was conducted after 7 days of processing. The standard concentration of nutrient solution were: Ca (NO_3_)_2_·4H_2_O, 826 mg/L; KNO_3_, 606 mg/L; NH_4_H_2_PO_4_, 114 mg/L; MgSO_4_·7H_2_O, 429 mg/L;Fe-EDTA, 7 mg/L; MnSO_4_·4H_2_O, 1.7 mg/L; Na_2_B_4_O_7_·10H_2_O, 2.45 mg/L; ZnSO_4_·7H_2_O, 1.45 mg/L; CuSO_4_·5H_2_O, 0.19 mg/L; Na_2_MoO_4_·2H_2_O, 0.12 mg/L. The concentration of N in the standard nutrient solution was 168 mg/L, and the concentration of K^+^ was 234 mg/L. The nutrient solution was applied once every morning from 8:00 to 9:00, with a dosage of 600 mL applied each time. The N treatment and K treatment were set at three levels: 50%, 100%, and 150%,, as shown in Table 1. The microstructure, surface morphology, cellulose and lignin content, puncture mechanical characteristics, and epidermal cell morphology of the cucumber stems were assessed, and micro-CT images were obtained.

**Table 1:** Nitrogen and potassium application levels

Treatment	Nitrogen (N)	Potassium (K)
Control group (CK)	100%	100%
Low K group (LK)	100%	50%
High K group (HK)	100%	150%
Low N group (LN)	50%	100%
Low N and low K group (LNLK)	50%	50%
Low N and high K group (LNHK)	50%	150%
High N group (HN)	150%	100%
High N and low K group (HNLK)	150%	50%
High N and high K group (HNHK)	150%	150%

### Stem microstructure

Since fixation and paraffin embedding solidify the internal tissue structure of cucumber stem, paraffin sectioning is often used for observing the true state of the internal tissue structure. Observation of the longitudinal profile of the cucumber stem helped elucidatethe tissue distribution characteristics along the puncture route and revealed the changes in the puncture mechanics curve.

### Stem surface morphology

On the day of inspection, the cucumber plants were moved to the laboratory after sufficient irrigation with water, and the stems were immediately observed after being picked to prevent water loss from affecting the test results. After the stem was cut along the central axis, it was placed under a super depth of field three-dimensional microscope (VHX-900F, KEYENCE Co., Japan) to observe the contour features and epidermal morphology of the stem, construct a three-dimensional image, and measure the size (μm). The ultra-depth of the field 3D microscope used a 1/1.8 type 1.95 million pixel CMOS image sensor with an effective pixel size of 1600 (H) × 1200 (V) and a maximum frame rate of 50 F/S. The ultra-depth of the field three-dimensional microscope and sample microscopic observation diagram are shown in Figure S1.

### Micro CT scan image of the stem

Micro-CT scanning technology, which can achieve sub-μM-level ultra-high resolution and exhibits other advantages such as non-destructive testing, rapid imaging, and 3D reconstruction, is widely used for obtaining microscopic 3D imaging information of plants [19, 20]. Therefore, this technique was used herein to obtain the true healing status of the stem wound after puncture. The micro-CT and stem wound measurements are shown in Figure S2.

**Fig. 1:**
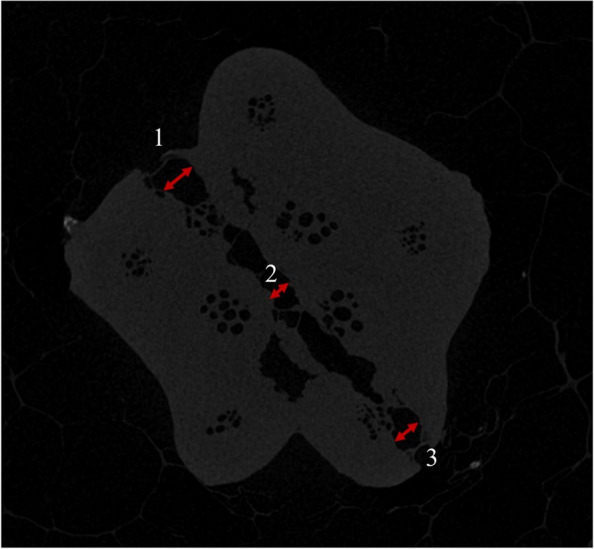
Micro CT and stem wound measurement. In the figure, 1, 2, and 3 are the initial, middle, and end positions of the puncture, respectively. The wound length is the average of the wound lengths at these three locations, mm

Fifty robust cucumber plants were selected and punctured using a probe with a diameter of 0.7 mm. After puncturing, five cucumber plants were randomly selected, and the internodes of the stems with puncture wounds were immediately intercepted and scanned with micro-CT (μCT100, SCANCO, Switzerland) to obtain the initial state information of the puncture wounds, recorded at time 0. Then, five cucumbers were randomly selected every 5 h, and the stem internodes with wounds were cut for micro-CT scanning to obtain dynamic healing information of the stem wounds. The average length of the wound at three locations was used as the length of the puncture wound, and the values are shown in Fig. 1. The maximum X-ray tube voltage of micro-CT-μCT100 is 90 kVp, and the focal diameter is 5–30 μm. The resolution is less than 4 μm.

### Cellulose and lignin

Cellulose and lignin are the main structural carbohydrates of the stem, and their content is related to the mechanical characteristics of the stem [14, 21]. A Fibertec M6 semi-automatic fiber analyzer (Foss, Denmark) was used to analyze the content of cellulose and lignin using chemicals C_12_H_25_SO_4_Na, Na_2_HPO_4_, C_19_H_42_BrN and H_2_SO_4_ [14], and the detection range of the machine was 0.1–100%.

### Puncture test

The puncture force of the stem can be predicted by measuring the force required to pierce the epidermis [22]. To reduce the impact of nighttime consumption on plants, the puncture test needs to be conducted after irrigation. Second, to reduce the impact of stem dehydration deformation caused by cutting, on-site sampling of the tested plants was conducted in the laboratory.

A texture analyzer (TA-XTPLUS, Stable Micro System, UK) was used to puncture the cucumber stem internodes to determine the puncture force of the cucumber stem in different treatment groups. The testing force accuracy of TA-XTPLUS was 0.0002%, with a movement range of 0.1–370 mm and a testing speed of 0.01–40 mm/s. Five plants were selected for each treatment, and each plant was repeatedly punctured three times. The pre-test speed was 5 mm·s^−1^, the penetration speed was 2 mm·s^−1^, the post-test speed was 5 mm·s^−1^, the minimum perception force was 5 g, and the puncture depth was 10 mm (ensuring puncturing of the stem). The puncture was made at the middle position of the internode to avoid the side branches at both ends of the internode, which may affect the puncture force. It was ensured that the puncture probe was facing the axis of the stem to avoid swaying due to deviation. The details of the stem puncture test are shown in Figure S3.

**Fig. 2:**
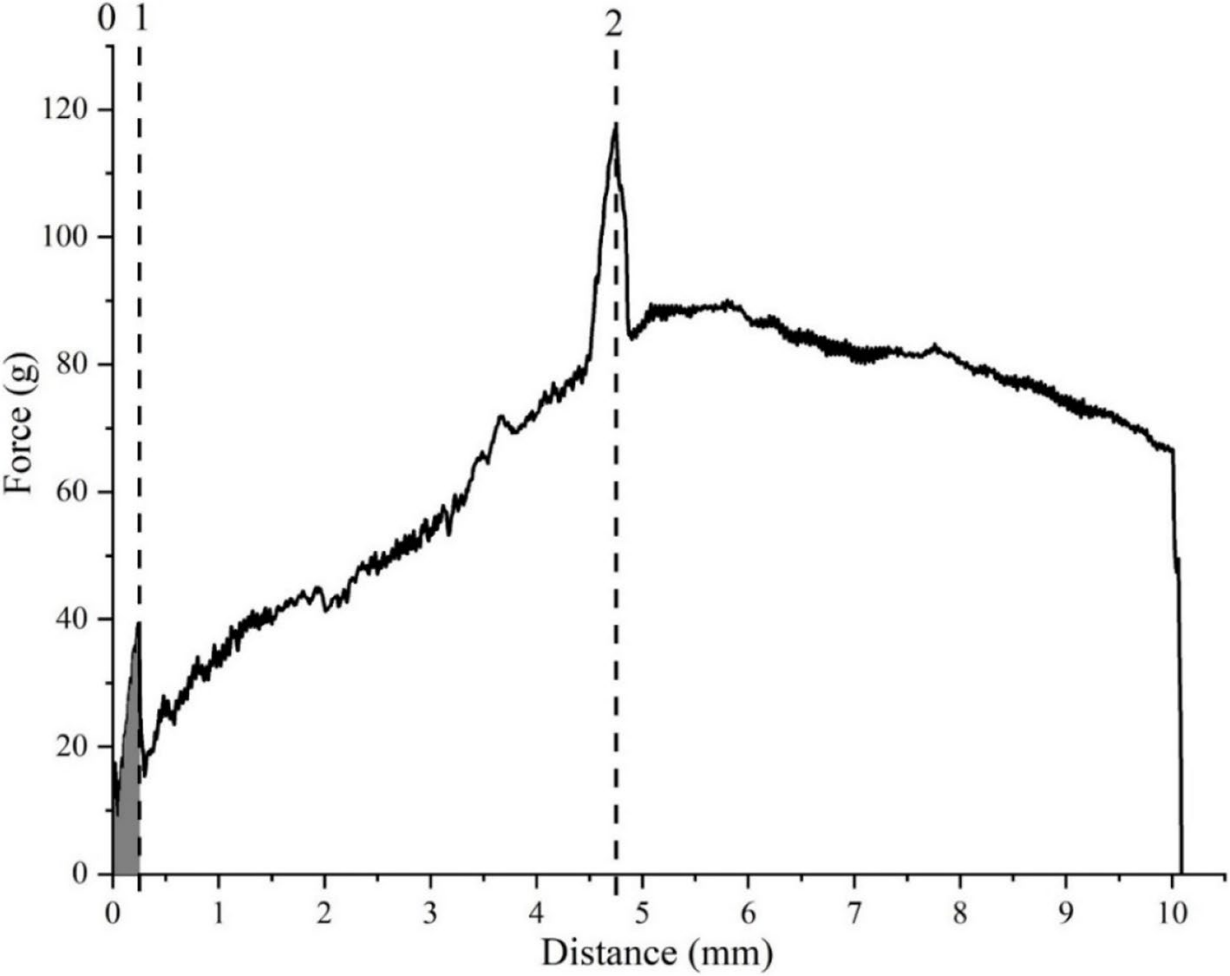
Puncture curve of greenhouse cucumber stem obtained using texture apparatus. The X-axis represents the movement distance of the puncture probe after contact with the epidermis, mm; the Y-axis represents the force perceived by the probe after contact with the epidermis, g

The classic force curve of the cucumber stem puncture obtained in the puncture experiments is shown in Fig. 2. Based on the results of previous puncture detection experiments [23, 24], the meaning of the obtained curve was defined. Among them, (1) was the first peak; (0–1) was the operating distance of the first peak; and (2) was the maximum peak value. The mechanical parameters for cucumber stem puncture can be defined as follows.

- Epidermal penetration force: The first peak of the puncture curve (1) represents the force required for the probe to penetrate the epidermis of the cucumber stems, defined as the epidermal penetration force (g).

- Epidermal break distance: The running distance of the first peak (0 to 1) represents the deformation of the cucumber stem epidermis before breaking, defined as the epidermal break distance (mm).

- Stem penetration force: As the puncture probe operates, the resistance continues to increase and pierces the other end of the epidermis at the second peak. The maximum peak (2) is the maximum penetration force of the stem, defined as the stem penetration force (g).

- Epidermal brittleness and epidermal toughness: We define the ratio of the first peak to its operating distance as epidermal brittleness (g·s^−1^), and toughness can be used to represent the ability of a material to absorb energy during plastic deformation and fracture processes, which can be expressed as the ratio of energy absorbed before fracture to volume. Because the volume of the epidermal puncture can be regarded as a point, we define the work done by the first peak force as epidermal toughness (g × s).

### Surface micromorphology of stem epidermal cells

In this experiment, a biological atomic force microscope (Dimension FastScan Bio, Bruker, Germany) was used to scan the surface microstructure of greenhouse cucumber stem epidermal cells. The scanning range of this biological atomic force microscope is 30 μm × 30 μm × 3.5 μm. The fastest scanning speed is 120 Hz, with a longitudinal resolution of 0.03 nm and a sample stage size of 14 cm × 14 cm × 5 cm. The SNL-10 surface morphology probe imaged the surface microstructure of the cucumber stem epidermal cells in tap mode to obtain the surface roughness of the epidermal cells. Each sample was repeatedly measured 10 times, and after each measurement, the probe landing point was shifted by 50 μm to avoid damage to the microstructure of the sample cell surface caused by repeated measurements. The testing results of the biological atomic force microscope are shown in Figure S4. The method flowchart of this study is shown in Figure S5.

### Statistical analysis

The mean values and standard deviations of each of the parameters studied including Cellulose and lignin content, surface roughness of stem epidermal cells, as well as stem mechanical characteristics were calculated. One-way analysis of variance (ANOVA) was performed for multiple comparisons. SPSS 18.0 was used for data analysis, and the least significant difference test (LSD) was used to determine significance at the level of *P* = 0.05.

## Results

### Microstructure and surface morphology of the stem

**Fig. 3:**
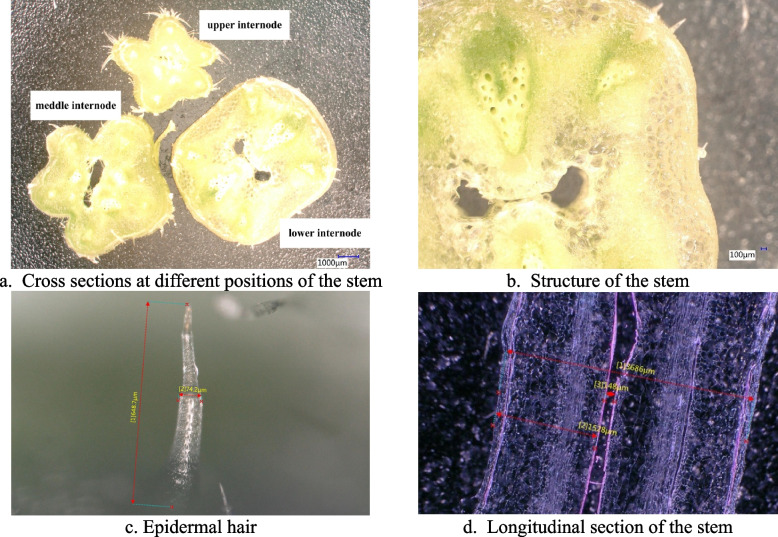
Microstructure and surface morphology of the cucumber stem. a is a cross section contour of different internodes of the stem; b is the distribution of the tissue structure of the stem; c is the size of the epidermal hairs, μm; and d is the longitudinal section of the stem. **a** Cross sections at different positions of the stem. **b** Structure of the stem. **c** Epidermal hair. **d** Longitudinal section of the stem

**Fig. 4:**
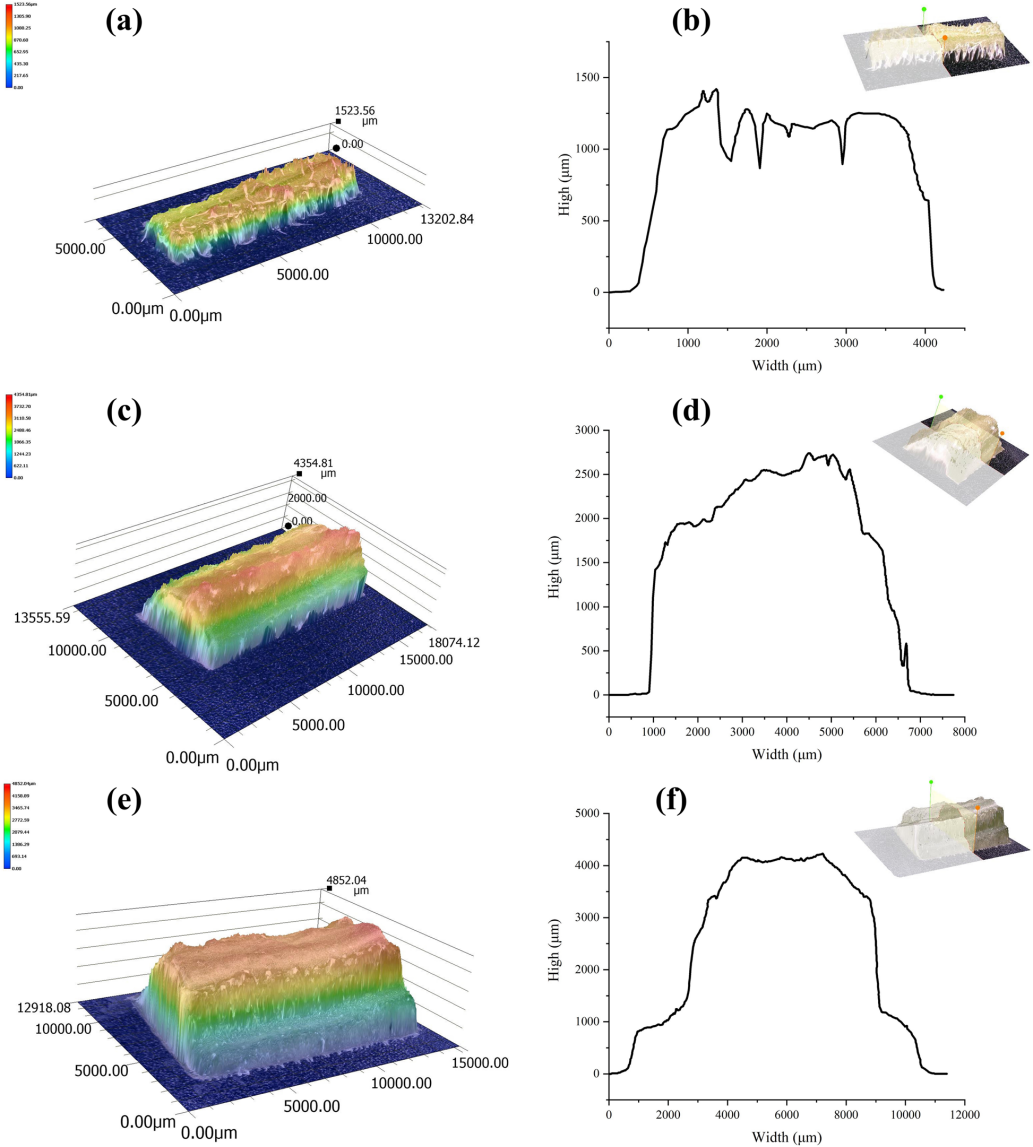
Surface morphology of the cucumber stem. a and b show the 3D images and surface morphology of the upper internodes; c and d show the 3D images and surface morphology of the meddle internode; and e and f show the 3D images and surface morphology of the lower internode. **a** Three-dimensional image of the upper internode. **b** Surface morphology of the upper internode. **c** Three-dimensional image of the middle internode. **d** Surface morphology of the meddle internode. **e** Three-dimensional image of the lower internode. f. Surface morphology of the lower internode

The microstructure and surface morphology of the cucumber stems are shown in Figs. 3 and 4. The newly grown tender stems had a large amount of transparent epidermal hairs on the surface, while the surface of the middle and lower stems had fewer hairs. the epidermal hair length was 253.2 – 648.7 μm. As shown in Fig. 4b, the diameter of the upper stem was approximately 4100 μm, and the height of the surface profile relative to the central axis was generally less than 1500 μm. Therefore, epidermal hair had a significant influence on the contour of the upper tender stem.

The shape of the stem was related to its height. The cross-section of the upper stem was starfish-shaped, and the randomness of edge development was strong. The surface was covered with a large amount of epidermal hairs, and the surface of the stem had dense and deep grooves. The physiological activity of the upper stem was the most vigorous, exhibiting the fastest growth rate and the highest sensitivity to changes in the external environment. However, the internode length and diameter were small, and the moisture content of the internal tissue was high, making it generally difficult to perform puncture analysis. Additionally, the epidermal hairs and edges of the upper stem interfered with the probe's perception of force during puncture testing.The lower stem had a cross-sectional area closer to a regular square or cylindrical shape compared with the upper tender stem, and the surface was smoother. Compared with the upper tender stem, the lower stem had a deeper degree of lignification, a thicker epidermis, and coloration that became gradually lighter, with the least physiological activity undertaken. Moreover, the lower stems began to develop first, and most of them had already completed biomass accumulation or structural construction before water and fertilizer treatments, resulting in small differences in their mechanical characteristics under different treatments.

The diameter of the middle stem was approximately 7000 μm, and compared with the upper stem, the number of edges and epidermal hairs decreased, the shape tended to be flat, and the number and depth of the surface grooves decreased. Therefore, the middle stem was less affected by epidermal hairs and contours, making it easier to perform puncture analysis. Compared with the lower stem, the middle stem had a lower regularity in shape, but its physiological activities were more vigorous. Biomass accumulation and structural development of the stem epidermis were still in progress, and the perception of N and K supply was also more sensitive. Therefore, the middle stem of the fourth to fifth internode at the top was ultimately used for the analysis of puncture mechanical characteristics.

### Healing time of the stem puncture wound

**Fig. 5:**
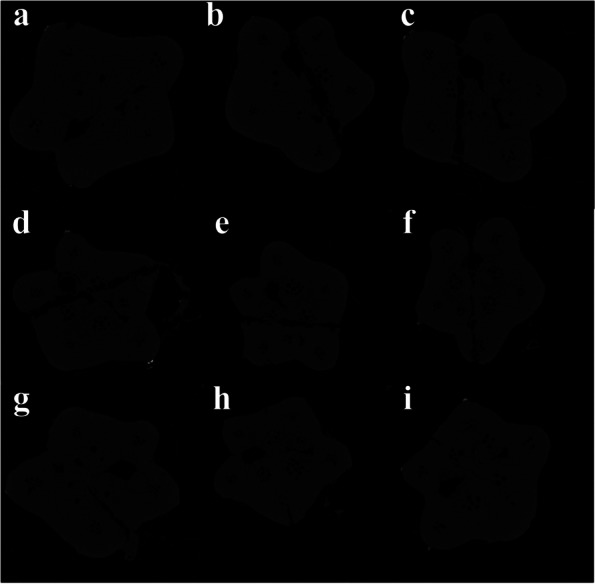
Micro-CT scan image of the stem wound: a shows the CT scan image before puncture; b shows the CT scan image at the time of puncture; and c–i shows the CT scan image every 5 h

The healing dynamics of the greenhouse cucumber stem puncture wounds in greenhouse are shown in Fig. 5. From Fig. 5c, it can be seen that the cucumber stem began to repair the wound damage within 5 h after puncturing.

According to Figs. 5e and 5f, it can be seen that there were obvious signs of healing inside the stem at 15 h after puncturing At 25 h after puncturing, the interior of the stem had approached the completion of repair; After 30 h, the repair and healing of the stem approached stagnation. This indicates that the entire repair process of the stem started with internal cells and structures and then gradually extended towards the edge and that the healing of the epidermal cells significantly lagged behind the repair of the stem interior.

**Fig. 6:**
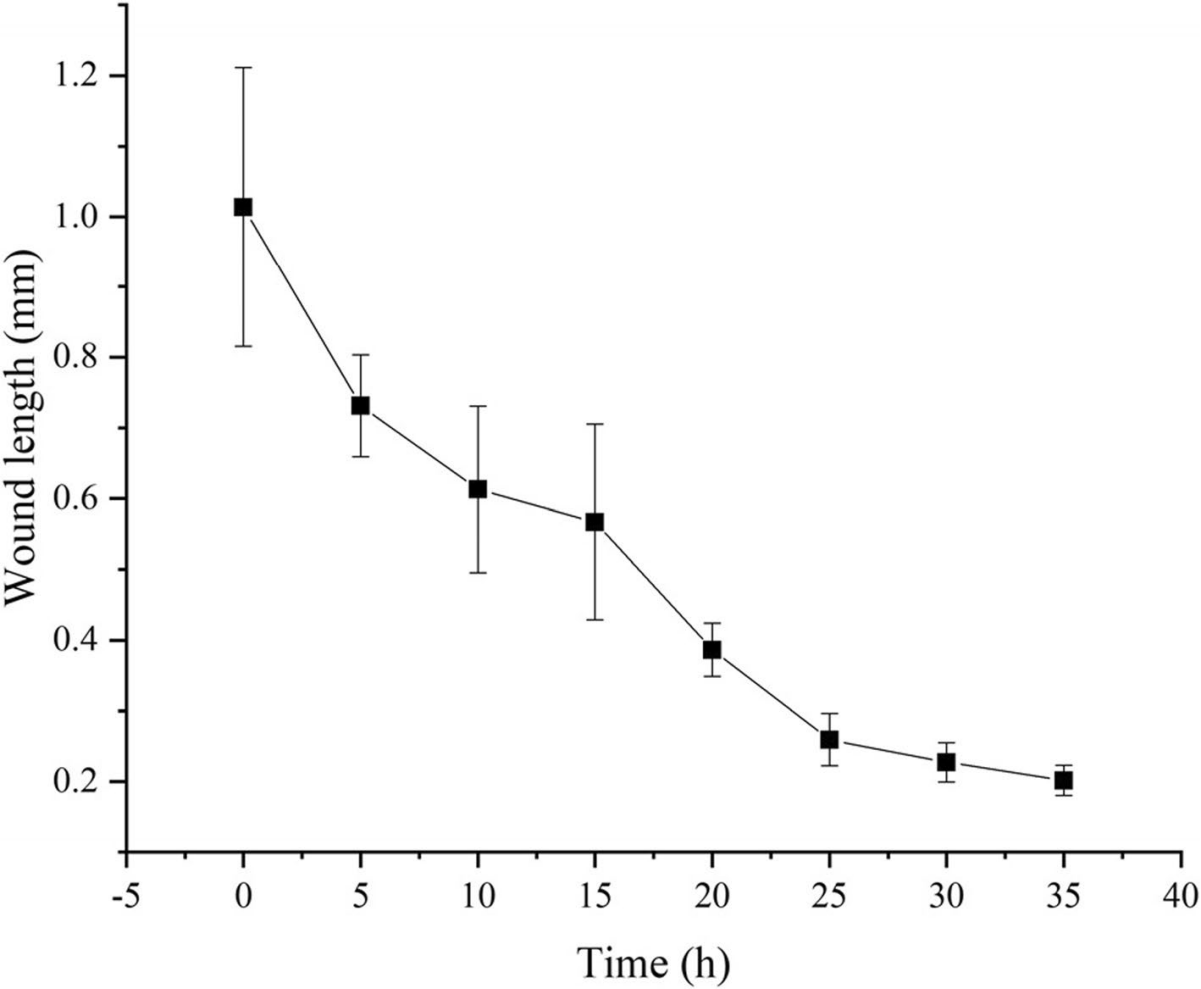
Healing curve of stem wound. The X-axis represents the time after puncture, h; the Y-axis represents the length of the puncture wound, mm

The healing curve of the cucumber stem puncture wound is shown in Fig. 6. It can be seen that within 5 h after puncturing, the length of the wound on the cucumber stem decreased from 1.01 mm to 0.73 mm, and then the healing speed slowed down. Between 15 and 25 h after puncturing, the length of the wound decreased from 0.57 mm to 0.39 mm, at which point the healing degree of the stem wound reached 74%. This indicates that during 0–5 h and 15–25 h, the speed of wound healing and repair was the fastest.

### Effect of N and K stress on the content of cellulose and lignin in the stem

According to previous research, the stem is mainly influenced by cellulose and lignin [21, 25], and N and K are key to their synthesis and numerous related physiological activities [26, 27]. Therefore, this study set up eight treatment groups and one control group (CK) of different the levels and combinations of N and K to investigate the changes in stem mechanical properties under different N and K stresses and the reasons for these changes.

**Fig. 7:**
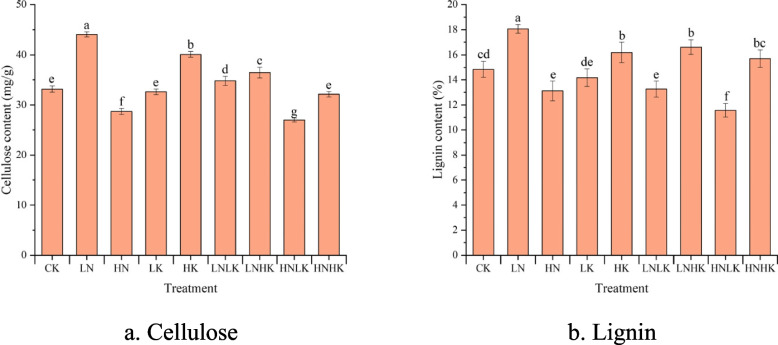
Changes in cellulose and lignin contents in the stems under different treatments. a is the cellulose content in the stem, g; b is the lignin content in the stem, g. Different superscript lowercase letters indicate a statistically significant difference in cellulose and lignin content in cucumber stems under different treatments at *P* = 0.05. a. Cellulose. b. Lignin

The changes in cellulose and lignin content in the stem under different treatments are shown in Fig. 7. Compared with CK, the cellulose and lignin in the stem increased by 32.9% and 21.7% under the LN treatment, but decreased by 13.39% and 11.62% under the HN treatment, respectively. Compared with CK, the content of cellulose and lignin significantly increased by over 9% under the HK treatment. Compared with CK treatment, the cellulose content under the LNLK treatment increased by 4.98%, while lignin decreased by 10.62%, and cellulose and lignin under the LNHK treatment increased by at least 10%. Under the HNLK treatment, cellulose and lignin decreased by 18.61% and 22.02%, respectively, in comparison with CK, while the lignin content increased by 5.75% under the HNHK treatment.

### Effect of N and K stress on the mechanical characteristics of the stems

**Fig. 8:**
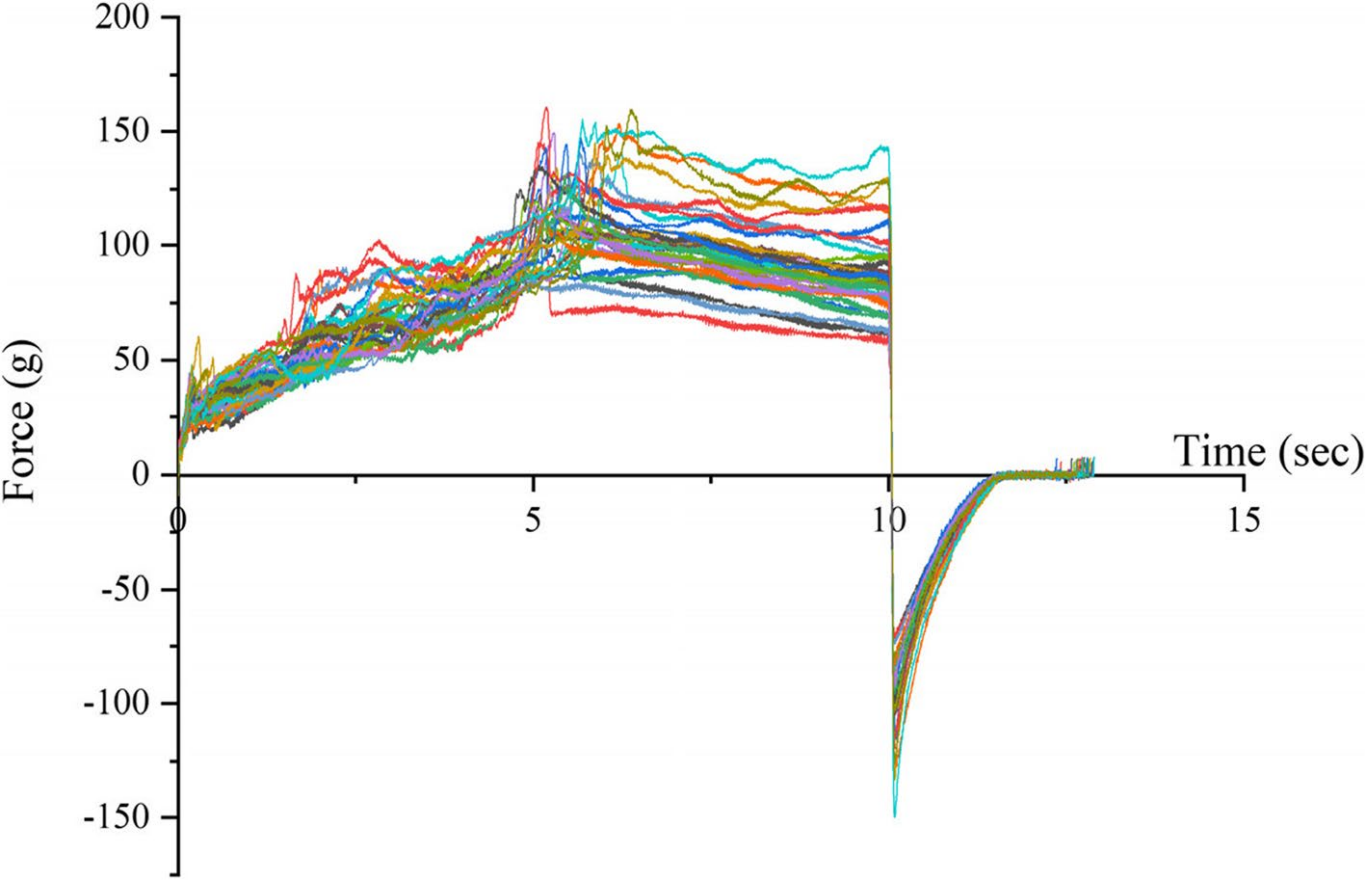
Changes in the mechanical characteristics of the stem under different treatments. The X-axis represents the movement time of the puncture probe after contact with the epidermis, s; the Y-axis represents the force perceived by the probe after contact with the epidermis, g

The puncture force time curve of the greenhouse cucumber stem puncture process is shown in Fig. 8. As shown in the figure, the entire puncture process was divided into three stages. The puncture force value in the first stage continuously increased until the first peak. This stage included the process of piercing the stem epidermis with a puncture probe. The puncture force in the second stage fluctuated slightly and gradually increased until just before the second peak. In the third stage, the puncture force rapidly increased, reached the second peak, and then suddenly decreased. This was because the puncture probe reached the other end of the epidermis and punctured it, and after puncturing it, the resistance disappeared, resulting in a rapid decrease in puncture force.

Although the variation trend of the puncture curve was roughly the same under different treatments, there were significant differences in the first peak, the distance of the first peak, and the maximum peak of the curve under different treatments. Therefore, we studied the changes in the mechanical characteristics of the stems under different N and K stresses.

**Fig. 9:**
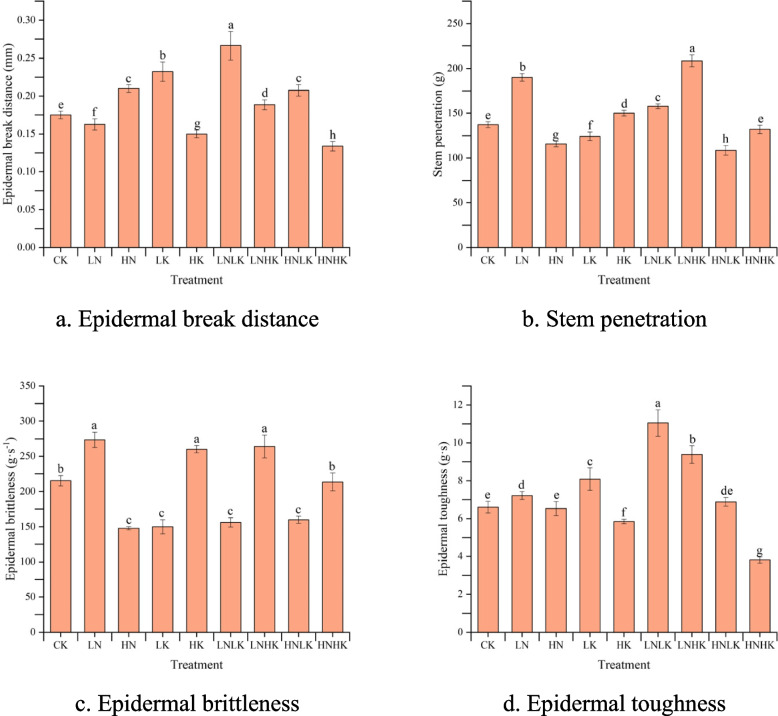
Changes in the mechanical characteristics of stem under different treatments: a shows epidermal break distance; b shows stem penetration; c shows epidermal brittleness; and d shows epidermal toughness. Different superscript lowercase letters indicate statistically significant differences in the epidermal break distance, stem penetration, epidermal brittleness, and epidermal toughness of cucumber stems under different treatments at *P* = 0.05. **a** Epidermal break distance. **b** Stem penetration. **c** Epidermal brittleness. **d** Epidermal toughness

The changes in the puncture mechanical characteristics of the cucumber stems under different N and K stresses are shown in Fig. 9. In accordance with previous research, in comparison with CK, the epidermal penetration of the LN, LNLK, and LNHK treatments increased by 17.77%, 10.08%, and 31.96%, respectively, while on the contrary, the values in the HN, LK, HNLK, and HNHK treatments decreased by 17.64%, 7.69%, 12.01%, and 24.34%, respectively [24]. The change trend in stem epidermal penetration was mainly influenced by the cellulose content inside the stem; that is, when the cellulose content inside the stem increased, the stem epidermal penetration also increased. Lignin had no decisive effect of stem epidermal penetration, for example, under the LNLK and HNHK treatments, the lignin content increased, but the epidermal penetration of the stem significantly decreased.

Compared with CK, the HN, LK, LNLK, LNHK, and HNLK treatments increased the epidermal break distance of the stems by at least 7%, while the LN, HK, and HNHK treatments decreased the epidermal break distance by 7.14%, 14.29%, and 23.57%, respectively. When the cucumber was subjected to independent stress, the change trend in the epidermal break distance under N and K stress was exactly the opposite; that is, the epidermal break distance increased with the increase in N application and decreased with the increase in K application. In addition, under the combined stress of a high concentration of K^+^ and K deficiency, the epidermal break distance of cucumber stems decreased with the increase of N application rate. This indicates that K^+^ had a more significant impact on the epidermal break distance of the cucumber stems compared with N.

The epidermal brittleness under the LN, HK, and LNHK treatments increased by at least 20% compared with the CK treatment, while the epidermal brittleness under the HN, LK, LNLK, and HNLK treatments decreased by at least 25%. Generally, a fracture that undergoes significant plastic deformation before fracturing is called a tough fracture. A fracture that does not occur or only has a small amount of macroscopic plastic deformation before fracturing is referred to as a brittle fracture. A brittle fracture is a sudden fracture without warning, resulting in weak resistance to external shocks and vibrations. The decrease in epidermal brittleness indicated an increase in the plastic deformation ability of the stem epidermis. Under the HN treatment, this may be due to a decrease in the content of cellulose and lignin in the stem, resulting in changes in the structure of the epidermal cell wall and an increase in the plastic deformation ability of the stem epidermis. Under K deficiency stress or combined K deficiency stress, the decrease in K^+^ concentration increased the gap between cell walls due to cell dehydration, thereby increasing the plastic deformation ability of the stem epidermis. This indicates that K^+^ had a very significant impact on epidermal fragility.

Compared with CK, the epidermal toughness of the greenhouse cucumber stems increased by 9.32%, 22.51%, 67.44%, and 42.12% under the LN, LK, LNLK, and LNHK treatments, respectively, while the epidermal toughness of the stems under the HK and HNHK treatments significantly decreased. Under the LN and LNHK treatments, the cellulose in the cucumber stems significantly increased, leading to an increase in the epidermal penetration force of the stem. Moreover, the rate of change in the epidermal penetration force of the stem was significantly higher than the range of changes in the epidermal break distance under these two treatments.Therefore, even when the LN and LNHK treatments reduced or slightly increased the epidermal break distance, the work required to pierce the epidermis still increased. Under the LK and LNLK treatments, the epidermal break distance of the stem increased by at least 32.71%, which was much greater than the change in the epidermal penetration force of the stem. Therefore, the epidermal toughness of the stem under the LK and LNLK treatments showed a characteristic change in the epidermal break distance, with a significant increase.

According to Fig. 9b, compared with CK, the penetration of the greenhouse cucumber stems was significantly increased under the LN, HK, and LNHK treatments, while the stem penetration was significantly reduced under the HN, LK, and HNLK treatments. According to the variation pattern of cellulose and lignin in Fig. 7, we found that as the content of cellulose and lignin increased, the penetration force of the cucumber stems also increased. In addition, there was no significant difference in the penetration of the stem compared with CK under the HNHK treatment, where cellulose increased while lignin decreased. This indicates that there were many factors affecting the penetration force of the stems, which might be jointly determined by factors such as structural carbohydrates, structural composition, cell gaps, and cell surface roughness.

Overall, the effect of N on the mechanical characteristics of the cucumber stems was achieved by changing the content of structural carbohydrates, which was also the main factor affecting the mechanical characteristics of the stems. Therefore, the changes in stem mechanical characteristics under combined stress were mainly characterized by symptoms of N stress. K^+^, on the contrary, mainly exerted its influence by changing the cell water content and intercellular space, and its effect on the mechanical characteristics of the stems was significantly weaker than the accumulation of structural carbohydrates.

### Changes in surface micromorphology of stem epidermal cells under N and K stress

**Fig. 10:**
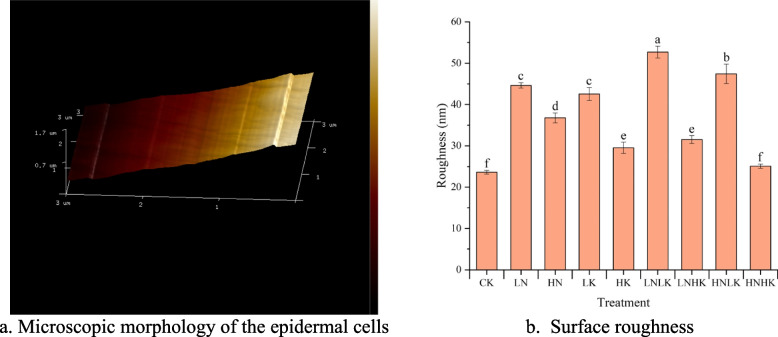
Surface characteristics of the epidermal cells of the cucumber stem. a is the microscopic morphology of epidermal cells; b is the surface roughness of epidermal cells of the cucumber stems. Different superscript lowercase letters indicate statistically significant differences in the surface roughness of the epidermal cells of the cucumber stems under different treatments at *P* = 0.05. **a** Microscopic morphology of the epidermal cells. **b** Surface roughness

Image of the cucumber stem epidermal cells scanned using atomic force microscopy are shown in Fig. 10. From Fig. 10b, it can be seen that except for the HNHK treatment, the surface roughness of the stem epidermal cells under the LN, HN,LK, HK, LNLK, LNHK and HNLK treatments increased by 88.56%, 55.71%, 79.72%, 24.97%, 122.99%, 33.43%, and 100.71% compared with CK, respectively. The significant increase in surface roughness of the stem epidermal cells under the LK, LNLK, and HNLK treatments indicated that when the concentration of K^+^ supplied from the outside was low, wrinkles or an uneven biomass distribution would form on the cell surface. The surface roughness of the stem epidermal cells under the HK, LNHK, and HNHK treatments was significantly lower, which was at the same level or slightly higher than that of the CK treatment. This indicates that a high K^+^ content reduced cell gaps, thereby promoting an increase in the surface smoothness of the epidermal cells.

## Discussion

The upper stem was most sensitive to external changes, but the internode length and diameter were small, and the epidermal hairs and edges interfered with the probe's perception of force, making it difficult to perform puncture analysis. The shape of the lower stem was regular, and the surface was flat, but the degree of lignification was high, and the biomass accumulation or structural construction had been basically completed, resulting in small differences in the mechanical characteristics of the stems under different treatments. The middle stem had a higher degree of regularity in shape than did the upper stem, making the puncture analysis easier than for the middle stem than for the upper stem.. Compared with the lower stem, the physiological activities were more vigorous, biomass accumulation and structural development were still ongoing, and the perception of element supply was more sensitive in the middle stem. Therefore, through a comprehensive analysis of factors such as morphological characteristics, physiological functions, and practical operations, the middle stem was ultimately selected for the analysis of puncture mechanical characteristics. Moreover, the middle stem showed a strong healing ability, almost completing healing within 25 h of being punctured. This is consistent with previous research [28]. Therefore, the puncture mechanical characteristics of the middle stem were used to detect nutritional stress in cucumbers, and as healing was achieved within 24 h, growth and development were unaffected.

Under single or combined stress, N played a more crucial role in the photosynthetic physiology, distribution of photosynthetic products, stress resistance, and mechanical characteristics of the stem epidermis compared with K^+^ [6]. Therefore, the changes in stem mechanical characteristics always exhibited obvious N stress characteristics. Related studies have shown that stem strength is mainly influenced by cellulose and lignin [21, 25]. Cellulose and lignin serves as structural carbohydrates in cell walls, and their relationship was similar to that of steel bars and cement in building construction. According to relevant research, cellulose in the stem formed the main structure of the cell wall, while lignin served as a filler to increase the compactness of the cell wall [29]. The cellulose and lignin content in the stem increased under the LN treatment, But decreased under the HN treatment, which was similar to the changes in the mechanical characteristics of the stem. The decrease in cellulose and lignin content affected the structure and strength of the stem cell walls. When the supply of K^+^ is insufficient, cells lose water to balance osmotic pressure, leading to an increase in intercellular space [30]. Therefore, under the LK treatment, the change in structural carbohydrate content within the stem was relatively small, but the penetration force of the stem epidermis decreased. Under the LNLK treatment, the cellulose content and epidermal penetration of the stem increased, but the lignin content significantly decreased. This indicates that cellulose had a more significant impact on epidermal penetration compared with ligninand that crops might also prioritize the synthesis of cellulose over lignin. This was also evidenced by the increase in lignin content and a decrease in epidermal penetration under the HNHK treatment.

Sucrose is the main transport form of photosynthetic products [31]. Research has shown that N deficiency and a high K^+^ concentration promot the outward transportation of sucrose [32, 33], and this is beneficial for the synthesis of lignin and cellulose in the stem. In addition, higher K^+^ promotes lignification of the stems [34]. Therefore, the cucumber stem strength (epidermal penetration and stem penetration) was improved under the LN and HK treatments. However, it was precisely because of the relief effect of K^+^ transport on osmotic pressure [35] that the changes in stem strength under the HK treatment were more gradual than those under the LN treatment. In addition, weaker connections between adjacent cells and instability of the cell structure are induced by lower K^+^ concentrations [36]. However, when crops perceive K deficiency, the mobilization mechanism of K^+^ is activated, which to some extent improves the efficiency of K^+^ absorption and utilization [37].

The HNHK treatment had higher lignin content compared with CK, but the stem epidermal penetration was lower. This may be because a higher N supply and K^+^ concentration promote cucumber metabolism, leading to an increase in the synthesis of lignin. However, the stem epidermal penetration was not the result of material accumulation, but was jointly determined by the content and composition of structural carbohydrates [22, 25, 29, 35]. Excessive K^+^ affects the absorption and utilization of N by crops, thereby inhibiting the formation of cell wall structures [38, 39] and reducing stem epidermal penetration.

When the lignin content in the stem decreased, the compactness of the cell wall decreased, leading to an increase in the break distance. Therefore, with the exception of the LNHK treatment, when the lignin content in the stem increased, the epidermal break distance decreased. Under the LNHK treatment, higher K^+^ concentrations not only promoted the lignification of the stem, but also encouraged the cells to absorb more water, thereby reducing cell gaps. This fully indicates that the changes in intercellular space caused by water had a more significant impact on the epidermal break distance compared with lignin content. The decrease in epidermal brittleness indicated an increase in the plastic deformation ability of the stem epidermis. Under K deficiency stress or combined K deficiency stress, the increase in plastic deformation ability was due to the decrease in K^+^ concentration, which increased the gap between cell walls due to cell dehydration. This indicates that K^+^ had a significant impact on epidermal brittleness. When the content of cellulose and lignin increased, the stem penetration increased. However, under the HNHK treatment where cellulose increased but lignin decreased, there was no significant difference in stem penetration compared with the CK treatment. This indicates that there were many factors influencing the penetration of the cucumber stems, which were jointly determined by factors such as structural carbohydrate content, structural composition, cell gaps, and cell surface roughness.

The powerful regulatory mechanism of crops is also reflected at the micro scale. During the growth and development of crops, physiological activities such as biomass synthesis and structural formation are selectively regulated based on environmental changes and the individual needs of plants [40, 41]. This might lead to stagnation in the process of cell wall formation in crops under stress. Therefore, under the LN treatment, a large amount of cellulose and lignin accumulated in the stem, but the surface smoothness of the stem epidermal cells still significantly decreased. In addition, when the concentration of K^+^ was high, the cells absorbed water and expanded, making the surface of the cells smoother [30, 42]. Therefore, the surface roughness of the treatments with higher K^+^ concentrations was comparatively lower than that of the other treatments.

## Conclusion

By analyzing the microstructure, healing rate, structural carbohydrate content, and changes in the mechanical characteristics of the stems, we elucidated the response of stem mechanical characteristics to N and K stress. The results indicated that the middle stem was less affected by epidermal hairs and contours compared with the upper and lower stems, and exhibited vigorous physiological activity, making it more sensitive to the perception of element supply. Therefore, the middle stem was selected for analysis of puncture mechanical characteristics. Moreover, the middle cucumber stems healed by 74% healing within 25 h, and their growth and development were therefore not affected by puncturing. The epidermal penetration of the cucumber stems was determined based on the content of cellulose and lignin, as well as the cell wall structure. Compared with lignin, cellulose had a more significant impact on the epidermal penetration of the cucumber stems and had a higher priority in crop regulation mechanisms. The epidermal break distance of the cucumber stems was mainly affected by the supply of K^+^. We found that the mechanical characteristics of the stems were a significant indicator of N application rate, but the detection effect of epidermal brittleness and toughness in multiple treatments was poor.

### Supplementary Information

**Additional file 1: Fig. S1. **Superdepth microscope and samples microscopic observation.** Fig. S2. **Micro CT and stem wound measurement. During scanning, put the stem sample into the container as shown in Figure S2-b and fix it with foam plate.** Fig. S3. **Puncture test with texture analyzer. **Fig. S4. **Biological atomic force microscope. **Fig. S5. **Method flowchart.

## Data Availability

The datasets used and/or analyzed during the current study are available from the corresponding author on reasonable request.
