# Supplementary material for: Preliminary study on the diagnosis of NK stress based on the puncture mechanical characteristics of cucumber stem
Source: BMC Plant Biol. 2024 Jan 3;24:26. doi: 10.1186/s12870-023-04675-0 (PMC10763222; doi:10.1186/s12870-023-04675-0)
Supplement: Supplementary file 1 — Additional file 1: Fig. S1. Superdepth microscope and samples microscopic observation. Fig. S2. Micro CT and stem wound measurement. During scanning, put the stem sample into the container as shown in Figure S2-b and fix it with foam plate. Fig. S3. Puncture test with texture analyzer. Fig. S4. Biological atomic force microscope. Fig. S5. Method flowchart. [file 12870_2023_4675_MOESM1_ESM.docx]

| 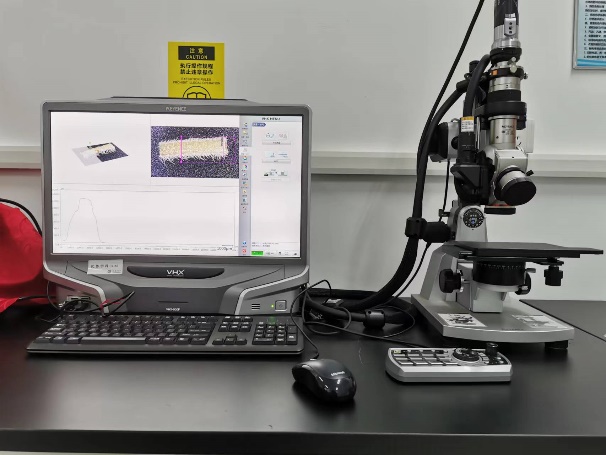 | 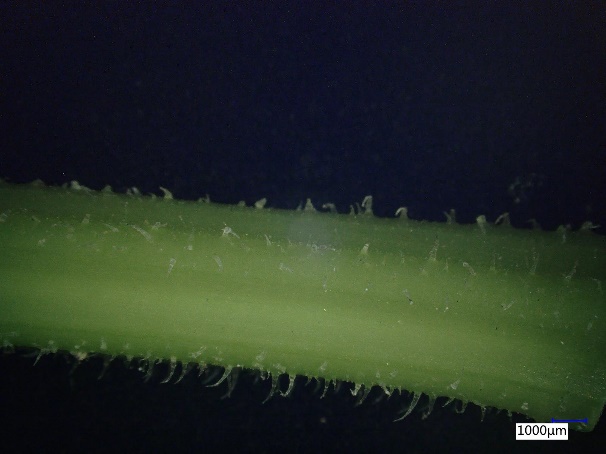 |
| --- | --- |
| a. VHX-900F | b. Surface morphology of stem |
| Fig. S1 Superdepth microscope and samples microscopic observation | |

| 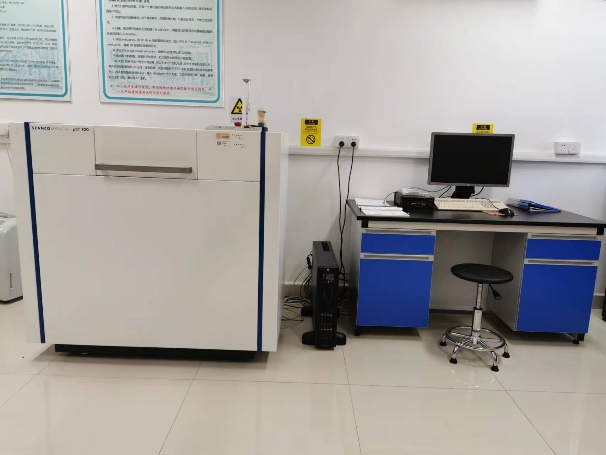 | 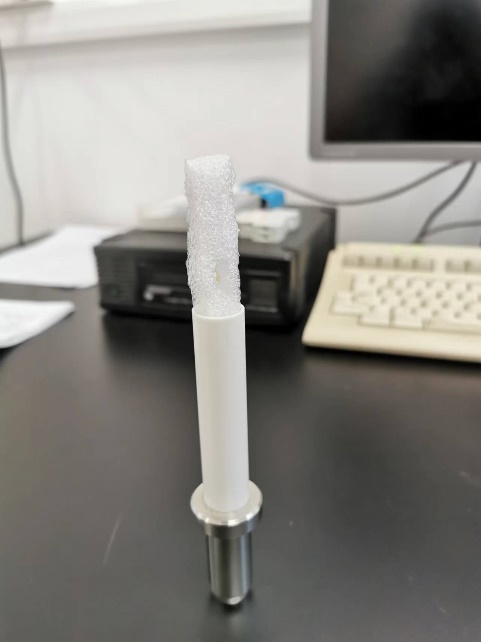 |
| --- | --- |
| a. Micro CT-μCT100 | b. Sample tube |
| 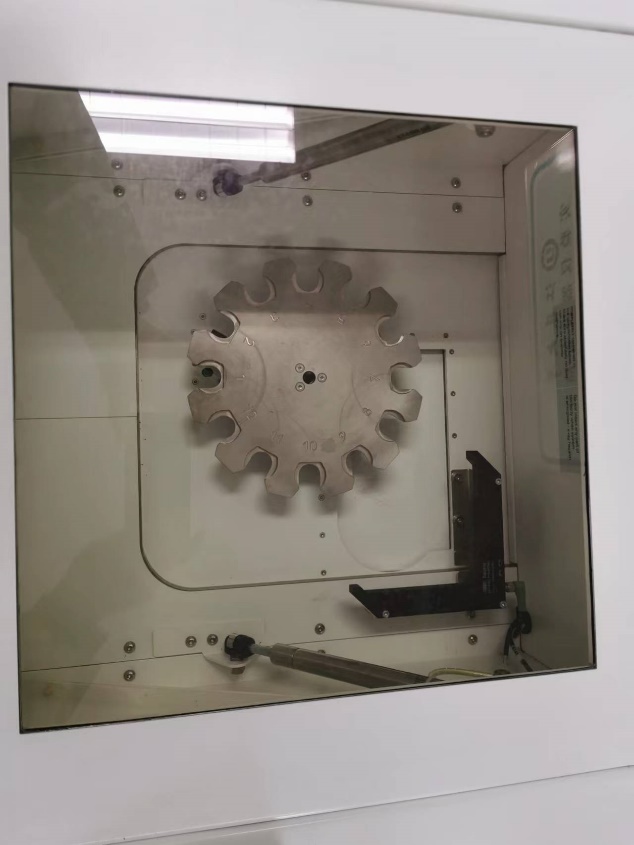 | |
| c. Sample room | |
| Fig. S2 Micro CT and stem wound measurement | |

During scanning, put the stem sample into the container as shown in Figure S2-b and fix it with foam plate.

| 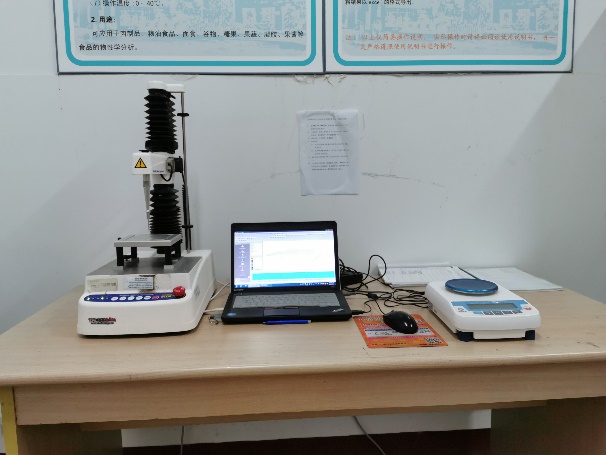 | 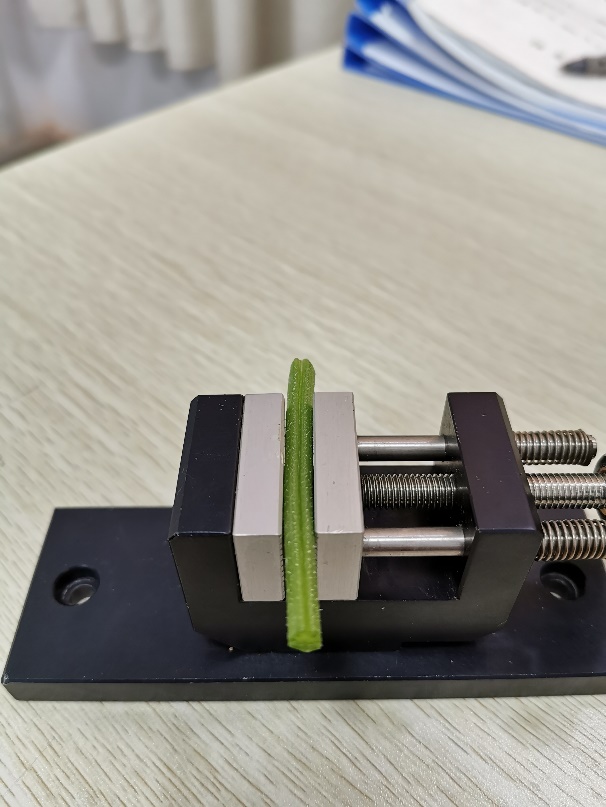 |
| --- | --- |
| a. TA-XTPLUS | b. Clamp |
| Fig. S3 Puncture test with texture analyzer | |

| 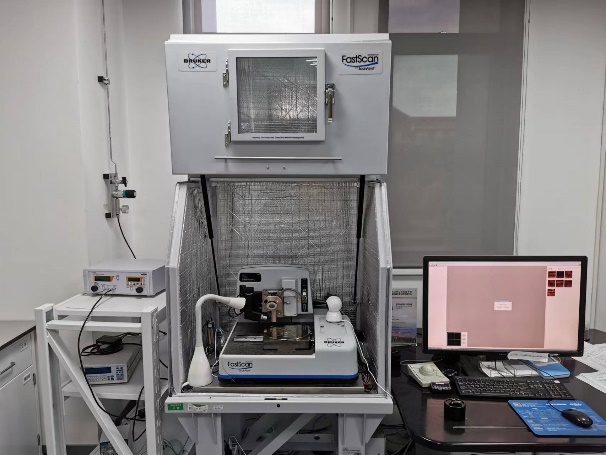 | 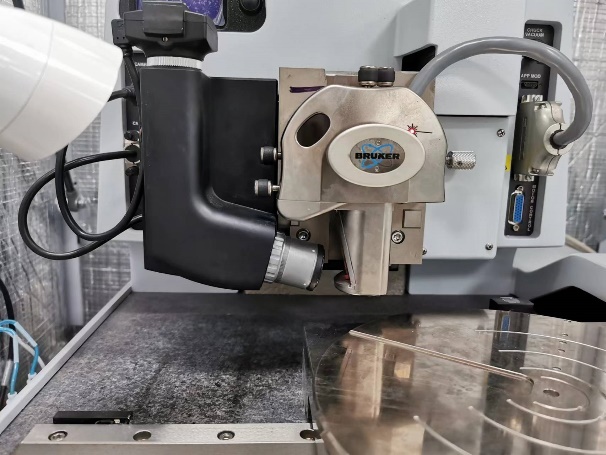 |
| --- | --- |
| a. Dimension FastScan Bio | b. Scan platform |
| 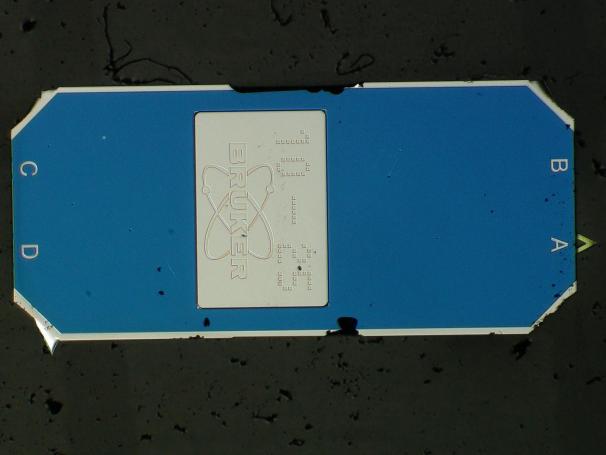 | 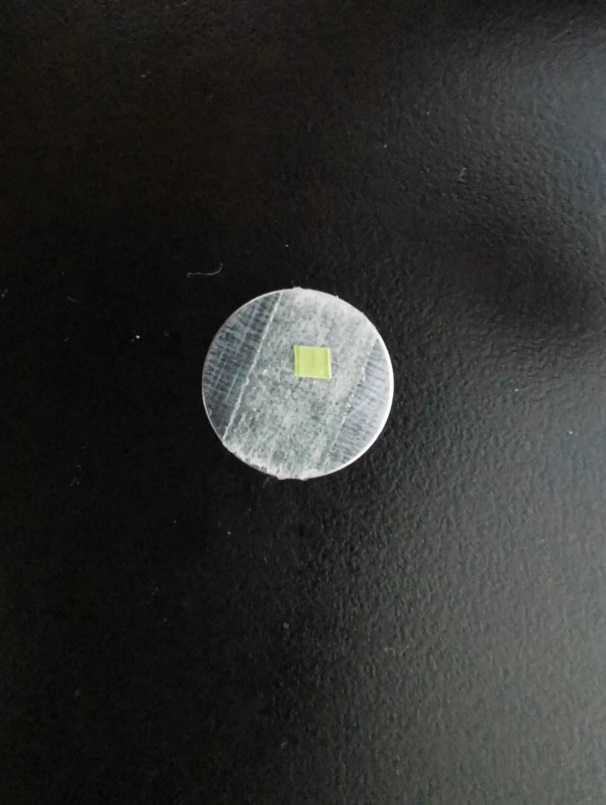 |
| c. SNL-10 probe | d. Sample to be tested |
| Fig. S4 Biological atomic force microscope | |

|  |
| --- |
| Fig. S5 Method flowchart |
